# Low levels of 3,3′-diindolylmethane activate estrogen receptor α and induce proliferation of breast cancer cells in the absence of estradiol

**DOI:** 10.1186/1471-2407-14-524

**Published:** 2014-07-21

**Authors:** Maud Marques, Liette Laflamme, Ines Benassou, Coumba Cissokho, Benoit Guillemette, Luc Gaudreau

**Affiliations:** 1 Département de Biologie, Université de Sherbrooke, J1K 2R1 Sherbrooke, QC, Canada

## Abstract

**Background:**

3,3′-diindolylmethane (DIM) is an acid-catalyzed dimer of idole-3-carbinol (I3C), a phytochemical found in cruciferous vegetables that include broccoli, Brussels sprouts and cabbage. DIM is an aryl hydrocarbon receptor (AhR) ligand and a potential anticancer agent, namely for the treatment of breast cancer. It is also advertised as a compound that regulates sex hormone homeostasis.

**Methods:**

Here we make use of RNA expression assays coupled to Chromatin Immunoprecipitation (ChIP) in breast cancer cell lines to study the effect of DIM on estrogen signaling. We further make use of growth assays, as well as fluorescence-activated cell sorting (FACS) assays, to monitor cell growth.

**Results:**

In this study, we report that ‘physiologically obtainable’ concentrations of DIM (10 μM) activate the estrogen receptor α (ERα) signaling pathway in the human breast cancer cell lines MCF7 and T47D, in a 17β-estradiol (E2)-independent manner. Accordingly, we observe induction of ERα target genes such as *GREB1* and *TFF1*, and an increase in cellular proliferation after treatment with 10 μM DIM in the absence of E2. By using an ERα specific inhibitor (ICI 182 780), we confirm that the transcriptional and proliferative effects of DIM treatment are mediated by ERα. We further show that the protein kinase A signaling pathway participates in DIM-mediated activation of ERα. In contrast, higher concentrations of DIM (e.g. 50 μM) have an opposite and expected effect on cells, which is to inhibit proliferation.

**Conclusions:**

We document an unexpected effect of DIM on cell proliferation, which is to stimulate growth by inducing the ERα signaling pathway. Importantly, this proliferative effect of DIM happens with potentially physiological concentrations that can be provided by the diet or by taking caplet supplements.

## Background

Breast cancer is one of the leading causes of death in industrialized countries and estrogens are known to play a role in its promotion [1]. Initiation of breast cancer by 17β-estradiol (E2) can involve the formation of DNA damage via its oxidation products. Accordingly, E2 is a substrate for the phase I cytochrome P450 (CYP) enzymes, CYP1A1 and CYP1B1. These two enzymes oxidize E2 into 2-hydroxyestradiol (2-OHE2) and 4-hydroxyestradiol (4-OHE2), respectively [2,3]. The 2-OHE2 metabolites can bind estrogen receptor α (ERα), but do not induce transcriptional activity [4]. On the other hand, 4-OHE2 hydroxylation results in the formation of a carcinogenic metabolite that can be further oxidized to highly reactive semiquinones and quinines [5]. These C-4 metabolites are well characterized and known to produce DNA adducts that lead to depurination of DNA [6-9]. CYP1B1 has been found in high concentrations in many types of tumors compared to normal tissues [10]. These observations suggest a function for CYP1B1 in promoting tumor growth. To support this hypothesis, the expression of *CYP1B1* has been observed in mammary tissue many weeks prior to the appearance of tumors in DMBA-treated rats [11]. Furthermore, in normal mammary tissue, 2-OHE2-derived metabolites are the main conversion products of E2, while a significant increase of 4-OHE2-derived metabolites is observed in cancerous mammary tissue. Based on these observations, a model has been put forth wherein the CYP1A1/CYP1B1 enzyme ratio is essential to control the intracellular level of genotoxic estrogen metabolites [12].

The *CYP1A1* and *CYP1B1* genes are expressed primarily in extra-hepatic tissue and are regulated by the aryl hydrocarbon receptor (AhR), a ligand-activated transcription factor that belongs to the bHLH/PAS family. AhR ligands are numerous and belong to several classes of chemicals including halogenated aromatic hydrocarbons (HAH) such as 2,3,7,8-tetrachlorodibenzo-p-dioxin (TCDD), polycyclic aromatic hydrocarbons (PAH) such as benzopyrene, and phytochemicals found in cruciferous vegetables like 3,3′-diindolylmethane (DIM). Female rodents exposed to TCDD for two years showed an increase in liver cancer incidence but a decrease in spontaneous mammary tumor formation [13]. Later studies revealed that TCDD and other AhR ligands inhibit cellular proliferation of human breast cancer cell lines, [14,15] as well as DMBA-induced mammary tumors in rats [16], and, consequently, these observations highlight a possible functional crosstalk between AhR and ERα signaling. The potential role of the AhR signaling pathway in mammary carcinogenesis inhibition led to the development of selective AhR modulators (SAhRMs) that act as potential anticancer agents. Even if TCDD possesses chemopreventive and chemotherapeutic proprieties in breast cancer development, it also induces acute liver toxicity. SAhRMs, like DIM, are reported to have the same inhibitory effects on mammary tumor formation in rats without having the deleterious effects of TCDD and other toxic AhR ligands. DIM is an acid-catalyzed dimer of indol-3-carbonyl (I3C), a compound found in cruciferous vegetables such as broccoli, Brussels sprouts and cabbage. DIM is one of the most biologically active products examined so far [17], and because of its potential chemotherapeutic functions, it has been extensively studied. Reports showed that DIM treatment induces a G1 arrest in the cell cycle of breast, ovarian, prostate, and colon cancer cell lines [18-23]. In addition, DIM also induces apoptosis and *p21* expression in a p53-independent manner [24-26], and is a low affinity ligand for AhR. However, conflicting reports can be found in the literature as to whether DIM is an agonist or an antagonist of AhR in the expression of the *CYP1* family of genes [27-31]. Furthermore, DIM activates ERα in a ligand-independent manner, which involves the protein kinase A (PKA) and mitogen-activated protein kinase (MAPK) signaling pathways under certain conditions [32].

As a natural compound, DIM can easily be taken as a dietary supplement. However, information regarding heavy DIM supplementation is scarce, and whether or not DIM use is safe on a long-term basis is not known. In this study, we compare the effects of two concentrations of DIM on the expression of AhR and ERα target genes, as well as test their impact on AhR-ERα crosstalk. We chose a lower concentration of DIM (10 μM; thereafter the ‘low concentration’), which can theoretically be reached in the human body by a ‘heavy eater’ of cruciferous vegetables, and a higher concentration (50 μM; thereafter the ‘high concentration’), which is known to possess strong anti-proliferative effects in cancer cells. Our results indicate an opposite dose-dependent effect of DIM in MCF7 and T47D cells in the absence of E2. At the high concentration, DIM inhibits cell proliferation and induces both *p21* and *CYP1A1* gene expression. At the low concentration, in the absence of E2, DIM acts as an estrogen mimetic and induces ERα target gene expression and concomitant cellular proliferation. Moreover, we find that the estrogenic effects observed following DIM treatment are mediated by ERα and the PKA signaling pathway.

## Methods

### Chemicals and reagents

2,3,7,8-Tetrachlorodibenzo-*p*-dioxin (TCDD) was obtained from Cerilliant Cambridge isotope Laboratories (catalogue #ED-901-C). 17β-Estradiol (E2) and ICI 182,780 (ICI) were purchased from Sigma-Aldrich. 3,3′-diindolylmethane (DIM) was purchased from LKT Laboratories, Inc. (catalogue #D3232), and H89 was purchased from Cayman chemical (catalogue #10010556).

### Cell culture and treatments

MCF7, T47D, and MDAMB-231 cell lines from American Type Culture Collection were maintained in DMEM (Wisent) containing 10% fetal bovine serum (FBS) and antibiotics. For all the experiments, cells were grown in phenol red free DMEM medium (Wisent) containing 5% dextran-coated charcoal-treated FBS and antibiotics for three days and then treated with different combinations of chemicals. For expression assays, we treated the cells for 24 h with 10 nM TCDD, 10 μM or 50 μM DIM, 100 nM E2. For ChIP assays, we treated cells with the same concentrations as described for the expression assays, but for 90 min with TCDD and TCDD + E2, and for 60 min with DIM and DIM + E2. In experiments with ICI H89, and PD98059, we added these chemicals 24 h prior to other treatments.

### RNA isolation and reverse transcription PCR

Cells were seeded in 6-well plates at a density of 0.35 × 10^6^ cells per well. The day after, the cells were washed twice with PBS and put in estrogen-free media for 3 days. The cells were incubated with ligands for 24 h. Total RNA was extracted from cells using Genelute (Sigma). cDNA was synthesized from 600 ng of total RNA using MMLV-RT (Promega).

### Quantitative real-time PCR

The synthesized cDNA was diluted to 1:8 and 5 μl of the dilution was used per reaction. Quantitative real-time PCR was performed using homemade 2X mix with SYBR Green, 2 mM MgCl_2_, and homemade Taq polymerase. We used qPCR primers for *36B4* as the internal control during qPCR. Human *CYP1A1*, *CYP1B1, GREB1, TFF1* and *p21* mRNAs were quantified with the following primers: RT *36B4* Fwd-CGACCTGGAAGTCCAACTAC; RT *36B4* Rev-ATCTGCTGCATCTGCTTG; RT *CYP1A1* Fwd-TGAACCCCAGGGTACAGAGA; RT *CYP1A1* Rev-GGCCTCCATATAGGGCAGAT; RT *CYP1B1* Fwd-AACGTACCGGCCACTATCAC; RT *CYP1B1* Rev-CCACGACCTGATCCAATTCT; RT *GREB1* Fwd-CGTTGGAAATGGAGACAAGG; RT *GREB1* Rev-CTCTGCCTGAAGGATGCTGT; RT *TFF1* Fwd-GTGCAAATAAGGGCTGCTGT; RT *TFF1* Rev-GCACATCCCTGCAGAAGTGT; RT *p21* Fwd-GGAGACTCTCAGGGTCGAAA; RT *p21* Rev-GGATTAGGGCTTCCTCTTGG.

### ChIP assays

ChIP assays were performed essentially as described previously [33]. Briefly, cells were crosslinked with 1.1% formaldehyde for 10 minutes and then quenched with 125 mM glycine. Samples were sonicated to generate chromatin fragments <500 bp. Next, the chromatin was immunoprecipitated with specific antibodies against AhR (SantaCruz) and ERα (SantaCruz). qPCR was performed using a set of primers relevant to the promoter regions of the *CYP1A1*. The primers used in qPCR are ChIP CYP1A1-A Fwd-CAGCACTAAGGCGATCCTAGA; ChIP CYP1A1-A Rev-GATTGAAGGATCGGAATGGA. Results are shown as percent of input.

### Cell proliferation assay

Cells were seeded in 48-well plates at a density of 1.5 × 10^4^ cells per well in estrogen-free media for three days and then treated with either DMSO; 100 nM E2; 10 μM DIM; 50 μM DIM; 50 μM ICI; 10 μM H89; 10 μM DIM and 50 μM ICI; or 10 μM DIM and 10 μM H89. Medium was replaced every two days. At each time point, cells were collected, fixed with 4% formaldehyde for 15 min, and kept in 0.4% formaldehyde/PBS 1X at 4°C until the last end point was reached. The cells were then washed once with sterile distilled water and colored with 0.5 mL of 0.1% crystal violet in 10% ethanol for 20 min. The cells were washed three times with sterile distilled water and allowed to air dry. The dye was extracted with 0.5 mL of 10% acetic acid for 20 min. Absorbance was measured at 590 nm in 96-well plates. The values are presented as fold over day 0. Each treated time-point is the average of nine wells from three independent experiments and the error bars represent standard deviation.

### FACS

Cells were rinsed with PBS, treated with trypsin and collected. The cells were then fixed in cold 70% ethanol, resuspended in 50 mM sodium citrate HCl pH 7.0, and treated successively with RNase A and proteinase K. Finally, the cells were resuspended in Sytox Green (Life Technologies) dye at a final concentration of 1 μM in the same buffer. Samples were analysed by flow cytometry on a Becton Dickinson FACScalibur cytometer. For each sample, ten thousand cells were plotted on a histogram with FL-1 on the X-axis and gates were set to distinguish cells in G1, S, G2/M and sub-G1-phases of the cell cycle. Data in the figure are expressed as a percentage of all gated cells in the sample and represent the average and standard error of triplicate experiments.

## Results

### Opposing effects of two AhR agonists on ERα-mediated repression of CYP1A1 expression

**Figure 1:**
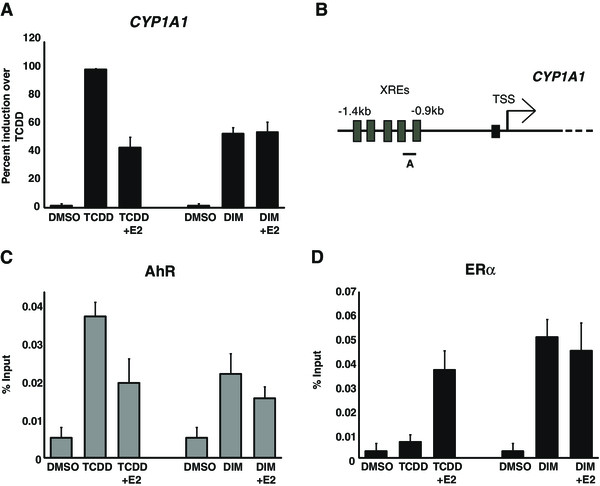
**DIM activates both AhR and ER signaling pathways. (A)** MCF7 cells, grown in estrogen-free media for three days, then treated with DMSO, 10nM TCDD, 10nM TCDD + 100nM E2, 50 μM DIM or 50 μM DIM + 100nM E2. After 24 h, the cells were lysed and RNA was extracted and quantified by RT-qPCR. Results are presented as percent induction over TCDD. **(B)** Schematic representation of the *CYP1A1* promoter and primer position used for ChIP analysis. ChIPs of AhR **(C)** and ERα **(D)** were performed in MCF7 cells, grown in estrogen-free media for three days, then treated with DMSO, 10nM TCDD, 10nM TCDD + 100nM E2, 50 μM DIM or 50 μM DIM + 100nM E2. Results are showed as% of Input and represent the mean of three independent experiments with standard deviation.

*CYP1A1* expression was first measured in MCF7 breast cancer cells that were grown in estrogen-free medium for three days and then treated with either 10 nM TCDD or 50 μM DIM, alone, or in combination with 100 nM E2 for 24 h. Co-treatment of cells with E2 and TCDD typically decreased *CYP1A1* activation by 60% when compared to TCDD alone (Figure 1A; [33]). Following DIM treatment, *CYP1A1* activity reached levels similar to those found in TCDD + E2 treated cells. Addition of E2 produced no effect on DIM-induced *CYP1A1* expression (Figure 1A). Similar results were also obtained in T47D cells, but we observed a more modest repression effect by E2, as well as weaker activation of *CYP1A1* (see Additional file 1: Figure S1A). We also monitored CYP1A1 protein levels upon TCDD treatment of cells, and, as expected, the observed effects on transcription correlated with CYP1A1 protein expression levels (Additional file 1: Figure S1B). To further investigate this phenomenon, we sought to verify the recruitment of AhR and ERα at the *CYP1A1* proximal promoter by ChIP experiments. As expected from the expression results, co-treatment of cells with TCDD and E2 impairs AhR binding at the *CYP1A1* promoter as compared to TCDD alone (Figure 1C). For the DIM treated cells, we observed no significant variation of AhR binding when E2 was added (Figure 1C). ERα recruitment to the *CYP1A1* promoter is known to occur only when AhR and ERα signaling pathways are simultaneously activated [34]. Our results are consistent with this, since ERα is present at the *CYP1A1* promoter only after treatment with TCDD + E2, but not after addition of TCDD alone (Figure 1D). Treatment of cells with DIM alone, or in combination with E2, resulted in the recruitment of both AhR (Figure 1C) and ERα (Figure 1D). This is consistent with the finding that DIM activates both AhR and ERα signaling pathways [30,35]. Altogether, these results show that *CYP1A1* expression and AhR and ERα binding at the *CYP1A1* promoter are differentially regulated by TCDD and DIM.

### Inhibition of ERα increases CYP1A1 induction in response to DIM

**Figure 2:**
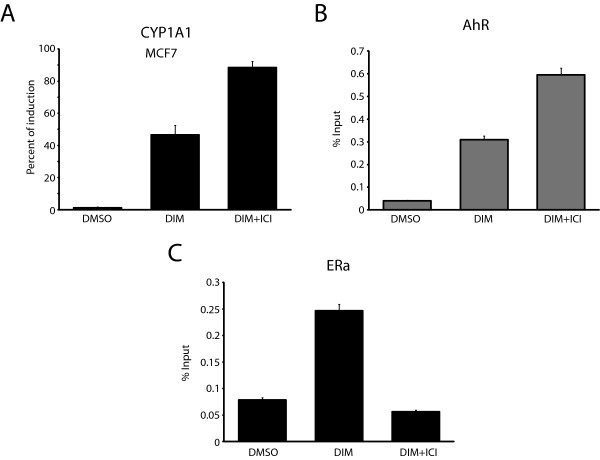
**ER**α **degradation increases*CYP1A1*induction in response to DIM. (A)** Expression analyses were performed in MCF7 cells grown in estrogen-free media for three days and treated with 50 μM ICI 182 780 for 24 h prior to the addition of 50 μM DIM for 24 h. ChIPs of AhR **(B)** and ERα **(C)** were performed in MCF7 cells, grown in estrogen-free media for three days and then treated or not with 50 μM ICI 182 780 for 24 h prior to the addition of 50 μM DIM for 1 h. Results are shown as% of Input and represent the mean of three independent experiments with standard deviation.

DIM is documented to be a weak AhR ligand when compared to TCDD and has been described as an antagonist of AhR-mediated gene transcription [30]. Considering the repressive effect of ERα on AhR signaling, the activation of ERα by DIM treatment might partly explain the weaker induction of *CYP1A1*. This scenario could also explain why co-treatment of cells with both TCDD and DIM leads to weaker induction of AhR target genes in ERα positive cells [30,36]. We next wanted to verify if depletion of ERα would allow an increase in *CYP1A1* expression after DIM treatment. First, MCF7 cells were grown in E2-depleted media for three days and then treated with 50 μM ICI 182 780 (a specific ERα inhibitor also known as Fulvestran) for 24 h prior to the addition of 50 μM DIM for another 24 h. We observed a two-fold increase in *CYP1A1* induction in cells treated with DIM and ICI compared to DIM alone (Figure 2A). A similar experiment was performed using T47D breast cancer cells and the results obtained (Additional file 2: Figure S2) were very comparable to those obtained in Figure 2A. ChIP experiments performed at the *CYP1A1* promoter using AhR and ERα antibodies show that AhR binding increases when cells are co-treated with DIM and ICI 182 780 (Figure 2B), and that ICI 182 780 prevents ERα from being recruited to *CYP1A1* (Figure 2C). The latter result is consistent with a previous study that showed that ICI leads to ERα degradation [37]. Overall, our data indicate a dual role of DIM in the regulation of *CYP1A1* expression. On one hand, DIM binds AhR and promotes *CYP1A1* induction, while on the other, DIM triggers ERα activation and represses *CYP1A1* expression.

### Different concentrations of DIM preferentially activate either the AhR or ERα signaling pathways

**Figure 3:**
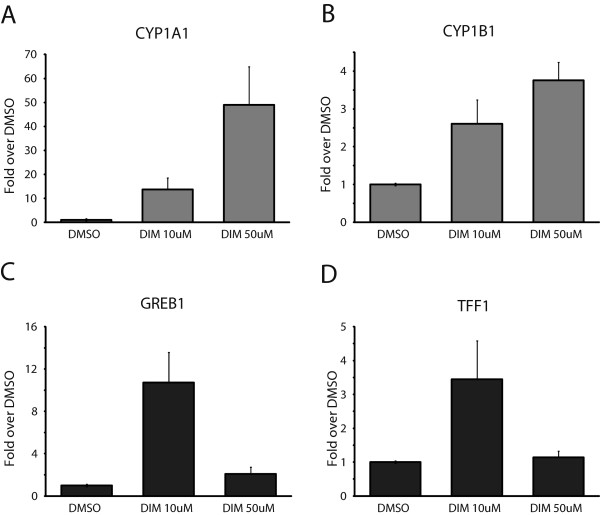
**Effects of different concentrations of DIM on AhR and ER**α **target gene expression.** mRNA levels of AhR target genes *CYP1A1***(A)** and *CYP1B1***(B)** and ERα target genes *GREB1***(C)** and *TFF1***(D)** were quantified in MCF7 cells grown in estrogen-free media for three days, then treated with DMSO, 10 μM DIM or 50 μM DIM for 24 h. Results are shown as fold over DMSO and represent the mean of three independent experiments with standard deviation.

In the experiments described above, we used 50 μM DIM, which is considered to be very high (the high concentration). For instance, Leong and co-workers proposed that a heavy eater of *Brassica* vegetables could reach, under optimal conditions, a DIM blood concentration of approximately 10 μM [35]. Thus, we decided to compare a potential physiological concentration of DIM (low concentration = 10 μM) with the high concentration (50 μM). We treated MCF7 cells grown in estrogen-free media for three days with the low and the high concentrations of DIM and then measured the mRNA levels of two AhR target genes (*CYP1A1* and *CYP1B1*), as well as two ERα target genes (*GREB1* and *TFF1*). We observed an increase in gene expression that is directly proportional to DIM concentrations for the AhR target genes (Figure 3A and B). Strikingly, the low concentration of DIM strongly induces ERα target gene expression, whereas the high concentration has almost no effect on the expression of these genes (Figure 3C and D). GREB1 protein levels were also monitored by immunoblotting using cells treated with 10 μM DIM (Additional file 3: Figure S3A). The results parallel the mRNA expression levels and show that *GREB1* is induced by 10 μM DIM. Taken together, our results suggest that physiological concentrations of DIM stimulate transcriptional activity of ERα-dependent genes in the absence of E2 in MCF7 cells. We also repeated these same experiments in T47D cells and obtained nearly identical results (Additional file 3: Figure S3B), a result that shows that these effects are not cell-type specific.

### The PKA signaling pathway contributes to DIM-mediated ligand-independent activation of ERα

**Figure 4:**
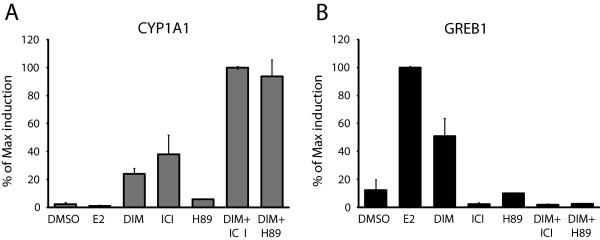
**DIM ligand-independent activation of ER**α **is mediated by the PKA signaling pathway.***CYP1A1* mRNA level **(A)** and *GREB1* mRNA level **(B)** in MCF7 cells grown in estrogen-free media for three days and then treated or not with 50 μM ICI or 10 μM H89 for 24 h prior to the addition of DMSO, 100nM E2, 10 μM DIM, 10 μM DIM + 50 μM ICI or 10 μM DIM + 10 μM H89 for 24 h. Results are shown as percent of maximum induction and represent the mean of three independent experiments with standard deviation.

A previous study using reporter assays has shown that the activation of ERα by DIM is independent of its binding to ERα and involves the PKA signaling pathway and, to a lesser extent, the MAPK pathway [32]. To test the role of the PKA signaling pathway in ERα activation by DIM, we used a specific inhibitor of the PKA pathway, H89. We measured mRNA levels of *CYP1A1*, which is negatively regulated by ERα, and *GREB1*, which is positively regulated by ERα. MCF7 cells grown in E2-depleted media were treated with either 100 nM E2, 10 μM DIM, 10 μM DIM + 50 μM ICI, or 10 μM DIM + 10 μM H89 for 24 h. Figure 4A shows that both the ICI and H89 treatments of cells abrogate the repression mediated by ERα on *CYP1A1* gene expression. Conversely, we observed that ICI and H89 abolish the induction of *GREB1* by DIM (Figure 4B). As with the previous figures, we performed the same experiments in T47D cells and obtained comparable results (Additional file 4: Figure S4). In conclusion, DIM mediates ERα activation, at least in large part, via the action of the PKA signaling pathway.

### Low concentrations of DIM induce MCF7 proliferation in the absence of E2

**Figure 5:**
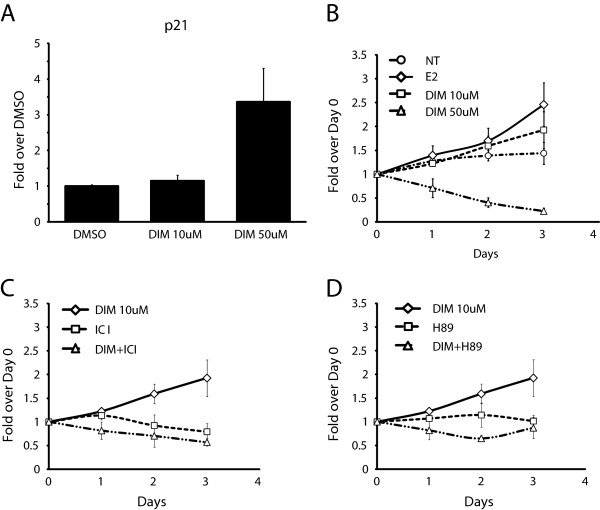
**Low concentration of DIM induces MCF7 proliferation in absence of E2. (A)***p21* expression was quantified in MCF7 cells grown in estrogen-free media for three days and then treated with DMSO, 10 μM DIM or 50 μM DIM for 24 h. **(B)** Proliferation assay of MCF7 cells, first grown in estrogen-free media and then treated (DMSO, ER, DIM 10 μM or 50 μM) over three days. **(C)** Proliferation assay of MCF7 cells treated with 10 μM DIM with or without ICI 182 780. **(D)** Proliferation assay of MCF7 cells treated with 10 μM DIM with or without H89.

It is known that high concentrations of DIM (>50 μM) have antiproliferative and antitumor properties in almost all cancer cell lines that have been tested [23,24,26]. Moreover, some of these properties have been proposed to work via induction of the *p21* gene, a key regulator of the cell cycle associated with G1 arrest and senescence [38]. Conversely, since a low dose of DIM activates ERα, it might also promote cellular proliferation. We thus decided to compare the effect of both DIM concentrations on cellular proliferation. We first verified the effect of high and low-dose DIM treatments on the expression of *p21* by RT-qPCR*.* We observe that only the high concentration of DIM induces *p21* expression in MCF7 cells (Figure 5A). We then compared MCF7 cell proliferation using crystal violet staining in E2-depleted media following three days of treatment with either E2, 10 μM DIM or 50 μM DIM (Figure 5B). Strikingly, the two concentrations of DIM have opposite effects on cellular proliferation. On the one hand, a low concentration of DIM stimulates cell growth almost as much as E2 treatment. On the other hand, a high concentration of DIM inhibits cell growth (Figure 5B). To verify that the observed effects of the low concentration of DIM on cellular proliferation were mediated by ERα and the PKA pathway, we treated MCF7 cells with either ICI or H89, in addition to DIM (Figures 5C, D). Both the degradation of ERα and the inhibition of the PKA signaling pathway abrogated the proliferative effect of DIM in the absence of E2. Similar experiments were conducted in T47D cells with comparable results (Additional file 5).

In order to further confirm the effects of DIM on the cell cycle, we performed cell cycle assays using flow cytometry (FACS) in T47D cells (Additional file 5: Figure S5B). The results show that at low concentrations of DIM (10 μM) the percentage of cells in S-phase is significantly increased compared to DMSO-treated cells, indicating a higher proliferation rate similar to cells treated with E2 (Additional file 6). Cells treated with 50 μM DIM tended to have a lower percentage of S-phase cells than untreated cells, although the difference was not statistically significant (*p*-value = 0.06). Finally, we performed a FACS experiment under the same conditions but with MDAMB-232 cells, which do not express the ERα. As expected, DIM has no significant effect (Additional file 6) on cell growth in this cell line, confirming that the proliferative effect of DIM is a result of activating the ERα pathway. In conclusion, we observed that treatment with the low concentration of DIM induced breast cancer cell proliferation in the absence of E2, an effect mediated by ERα and the PKA signaling pathway.

## Discussion

Bidirectional inhibitory crosstalk between AhR and ERα is very complex and occurs at many regulatory levels [39,40]. AhR ligands have been shown to carry potentially important chemopreventive properties, thus understanding the mechanisms behind these properties is fundamental for developing cancer therapies. DIM has been intensely studied as a possible therapeutic agent in cancer treatment, especially for breast cancer. Studies report that DIM treatment promotes cellular growth arrest of cancer cells, as well as a decrease in mammary tumor formation in DMBA-treated rats [16,24,30]. Although the use of DIM as a therapeutic agent in the treatment of breast cancer is not yet approved, there are active clinical trials that are testing DIM for the treatment of many types of cancers (http://clinicaltrials.gov/ct2/results?term=diindolylmethane). However, DIM can easily be purchased as a dietary supplement and be self-administered. As previously mentioned, DIM is a SAhRM that binds AhR, which is involved in the regulation of the expression of phase I and II drug metabolizing enzymes. Discrepancies are found in the literature as to whether DIM is an agonist or an antagonist of AhR [27-31], thus, clarification of this issue is important, especially regarding the potentially toxic effect mediated by AhR agonists in the liver following AhR activation.

In this study, we tested how the use of different concentrations of DIM can lead to opposite biological outcomes. As previously reported, we confirmed that activation of ERα by E2 represses the induction of *CYP1A1* by approximately 60% after TCDD treatment. The simultaneous activation of AhR and ERα when cells are treated with DIM does not allow full induction of *CYP1A1*. Furthermore, addition of E2 to DIM-treated cells has no repressive effect on *CYP1A1* expression, which can be explained by the fact that ERα is already fully recruited to the *CYP1A1* promoter after DIM treatment alone. We propose that activation of ERα by DIM can explain, at least in part, some discrepancies found in the literature on the role of DIM as an agonist/antagonist of AhR in ERα positive cell lines [27-31].

DIM concentrations found in the human body are dependent on the diet. Our first experiments were carried out using a concentration of 50 μM, which is probably much higher than what can realistically be reached in the body [35]. We then compared 50 μM DIM with a more physiological concentration of DIM (10 μM) and observed that the high concentration of the compound induces the expression of AhR target genes (*CYP1A1* and *CYP1B1*)*,* while the low concentration shows significant effects on the expression of ERα target genes (*GREB1* and *TFF1*) in the absence of E2. These observations indicate that at physiological concentrations, DIM principally mediates estrogenic effects. It can also explain why oral administration of DIM in rodents has no hepatic toxicity due to the weak induction of the *CYP1A1* gene at this low concentration. ERα activation can be mediated by direct binding of its main ligand (E2), but it can also be induced by the activation of the PKA signaling pathway. The phosphorylation of ERα increases its capacity to interact with the transcription machinery and triggers the expression of ERα target genes [41-44]. Accordingly, we were able to demonstrate that the effect of DIM treatment on *CYP1A1* and *GREB1* expression is mediated by ERα, which, in this case, is activated mostly by the PKA signaling pathway.

## Conclusions

The estrogen receptor is highly expressed in almost 70% of breast cancer cases and its activation promotes cellular proliferation and tumor development [45]. Our results demonstrate that DIM, at concentrations likely attainable by a diet rich in cruciferous vegetables, induces proliferation of MCF7 and T47D breast cancer cells in the absence of E2. DIM requires that ERα be activated by the PKA signaling pathway to promote cellular growth in the absence of E2. Consequently, the abundance of ERα, as well as circulating estrogen levels, will influence the local effects of DIM on cell growth. Altogether, our findings suggest that the use of DIM as a dietary supplement or as a therapeutic agent should be undertaken very cautiously as unexpected adverse effects could be encountered.

## Competing interests

The authors declare that they have no competing interests.

## Authors’ contributions

MM, LL, IB, CC, and BG performed the experiments. LG and MM conceived the experiments and wrote the manuscript. All authors read and approved the final manuscript.

## Pre-publication history

The pre-publication history for this paper can be accessed here:

http://www.biomedcentral.com/1471-2407/14/524/prepub

## Supplementary Material

Additional file 1: Figure S1 **(A)** DIM activates both AhR and ER signaling pathways in T47D cells. Cells were grown in estrogen free media for 3 days, then treated with DMSO, 10nM TCDD, 10nM TCDD + 100nM E2, 50 μM DIM or 50 μM DIM + 100nM E2. After 24 h, the cells were lysed, RNA was extracted and reverse transcribed. Results are presented as percent induction over TCDD. Results represent the mean of 3 independent experiments with standard deviation. **(B)** CYP1A1 protein levels are induced by treating cells with 10 μM DIM. Immunoblot of CYP1A1 using an anti-CYP1A1 antibody in T47D cells. Conditions are as described in **(A)** using 10 μM DIM or DMSO-treated cells. Click here for file

Additional file 2: Figure S2 ERα degradation increases *CYP1A1* induction in response to DIM. Expression analysis were performed in T47D cells grown in estrogen free media for 3 days and treated with 50uM ICI 182 780 for 24 h prior addition of 50uM DIM for 24 h. Results represent the mean of 3 independent experiments with standard deviation. Click here for file

Additional file 3: Figure S3 Effects of different concentrations of DIM on AhR and ERα target gene expressions. Protein levels of GREB1 are monitored by immunoblotting **(A)**, mRNA levels of AhR target genes *CYP1A1***(B)** and *CYP1B1***(C)** and ERα target genes *GREB1***(D)** and *TFF1***(E)** were quantified in T47D cells grown in estrogen free media for 3 days, then treated with DMSO, 10uM DIM or 50uM DIM for 24 h. Results are showed as fold over DMSO and represent the mean of 3 independent experiments with standard deviation. Click here for file

Additional file 4: Figure S4 DIM ligand-independent activation of ERα is not mediated by the PKA signaling pathway in T47D cells. *CYP1A1* mRNA level **(A)** and *GREB1* mRNA level **(B)** in T47D cells grown in estrogen free media for 3 days, and then treated or not with 50uM ICI or 10uM H89 for 24 h prior addition of DMSO, 100nM E2, 10 μM DIM, 10 μM DIM + 50 μM ICI or 10 μM DIM + 10 μM H89 for 24 h. Results are showed as percent of maximum induction and represent the mean of 3 independent experiments with standard deviation. Click here for file

Additional file 5: Figure S5 Low concentration of DIM induces T47D proliferation in the absence of E2. **(A)***p21* expression was quantified in T47D cells grown in estrogen free media for 3 days and then treated with DMSO, 10 μM DIM and 50 μM DIM for 24 h. Proliferation of T47D cells, grown in estrogen free media, was analyzed following various treatments during 3 days. **(B)** Comparison of T47D cell proliferation after DMSO, 100nM E2, 10 μM DIM and 50 μM DIM treatments. **(C)** Effect of ICI 182 780 on T47D cell proliferation induced by 10 μM DIM treatment. Click here for file

Additional file 6: Figure S6 FACS analysis of cells treated with either 10 or 50 μM DIM. **(A)** T47D or **(B)** MDAMB-231 cells. Bar plot shows the percentage of S-phase cells in each sample. Click here for file
